# Polyhydroxy Fullerenes Enhance Antibacterial and Electrocatalytic Activity of Silver Nanoparticles

**DOI:** 10.3390/nano12193321

**Published:** 2022-09-23

**Authors:** Luis Palomino, Danae A. Chipoco Haro, Miguel Gakiya-Teruya, Feng Zhou, Adolfo La Rosa-Toro, Vijay Krishna, Juan Carlos F. Rodriguez-Reyes

**Affiliations:** 1 Laboratory of Nanoscience and Applications—NASCA, Universidad de Ingenieria y Tecnologia—UTEC, 165 Medrano Silva, Barranco, Lima 15063, Peru; 2 Centro de Investigacion en Bioingenieria—BIO, Universidad de Ingenieria y Tecnologia—UTEC, 165 Medrano Silva, Barranco, Lima 15063, Peru; 3 Department of Biomedical Engineering, Cleveland Clinic, Lerner Research Institute, Cleveland, OH 44106, USA; 4 Laboratorio de Investigacion de Electroquimica Aplicada, Facultad de Ciencias, Universidad Nacional de Ingenieria, Av. Tupac Amaru 210, Rimac, Lima 15333, Peru; 5 Department of Chemical Engineering, Universidad de Ingeniería y Tecnología—UTEC, 165 Medrano Silva, Barranco, Lima 15063, Peru

**Keywords:** silver nanoparticles, polyhydroxy fullerene, antibacterial activity, oxygen reduction reaction, oxygen evolution reaction, one-pot synthesis

## Abstract

Silver nanoparticles (AgNPs) are known and widely used for their antibacterial properties. However, the ever-increasing resistance of microorganisms compels the design of novel nanomaterials which are able to surpass their capabilities. Herein, we synthesized silver nanoparticles using, for the first time, polyhydroxy fullerene (PHF) as a reducing and capping agent, through a one-pot synthesis method. The resulting nanoparticles (PHF-AgNPs) were compared to AgNPs that were synthesized using sodium citrate (citrate-AgNPs). They were characterized using high-resolution transmission electron microscopy (HR-TEM), dynamic light scattering, and UV-visible spectroscopy. Our results showed that PHF-AgNPs have a smaller size and a narrower size distribution than citrate-AgNPs, which suggests that PHF may be a better capping agent than citrate. Antibacterial assays using *E. coli* showed enhanced antimicrobial activity for PHF-AgNPs compared to citrate-AgNPs. The electrocatalytic activity of nanoparticles towards oxygen evolution and reduction reaction (OER and ORR, respectively) was tested through cyclic voltammetry. Both nanoparticles are found to promote OER and ORR, but PHF-AgNPs showed a significant increase in activity with respect to citrate-AgNPs. Thus, our results demonstrate that the properties of forming nanoparticles can be tuned by choosing the appropriate reducing/capping agent. Specifically, this suggests that PHF-AgNPs can find potential applications for both catalytic and biomedical applications.

## 1. Introduction

### 1.1. Metallic Nanoparticles Synthesis: Reducing and Stabilizing Agents

Metallic nanoparticles (MNPs) possess optical, electronic, and mechanical properties that differ from those found in the macroscopic scale [1]. These properties turn MNPs into materials with potential applications in several fields. For example, silver nanoparticles (AgNPs) have been used in drug delivery, catalysis, and water disinfection [2]. Diverse methods have been proposed for the synthesis of MNPs, such as chemical reduction, photochemical reduction, reverse micelle-based, and lamellar liquid crystal approaches [3]. In the chemical reduction method, the metal ions react with a reducing agent and form nuclei, which grow up to the nanoscale. Reducing agents for the chemical reduction method include glucose, hydrazine, citrate, and sodium borohydride, among others [4]. A strong reducing agent (i.e., borohydride) may hinder the generation of large-sized AgNPs, while a weaker one (i.e., citrate) allows for better control of size and shape due to the slower reaction rate [4,5]. This highlights the importance of an appropriate reducing agent for a specific application.

In addition to reducing agents, stabilizing agents are needed to adsorb on the surface of growing nanoparticles and to avoid particle growth, either through electrostatic repulsion or steric hindrance. Stabilizing agents or capping agents also help in controlling the size of the nanoparticles. Several ions and compounds, such as citrate ions, can be used simultaneously as reducing/stabilizing agents. Organic molecules such as alcohols and aldehydes can also be employed. Interestingly, due to its ease for reduction, silver nanoparticles can even be obtained using plant extracts containing some of the organic species mentioned above [6]. In addition to the nature of reducing and stabilizing agents, the conditions employed during nanoparticle syntheses directly affect their shape and size, and thus their electronic and chemical properties [4,6]. Different chemical reduction methods are available in the literature; we have recently provided protocols for two chemical reduction methods to synthesize AgNPs [7].

### 1.2. Antimicrobial and Electrocatalytic Properties of AgNPs

Silver nanoparticles are well known antimicrobial agents, operating through multiple mechanisms of action, including binding to sulfur-containing proteins, disrupting cell membranes, and promoting the generation of reactive oxygen species (ROS) [8,9,10]. Stabilizing agents can affect the antimicrobial activity of AgNPs. This could be due either to the reduced electron transfer process or to variations in particle size, with smaller particle sizes exhibiting higher antimicrobial activity [11,12]. In recent years, antimicrobial activity of AgNPs has been broadly studied for several microorganisms [13,14,15], which demonstrates the versatility of these nanoparticles as antimicrobial agents.

Silver nanoparticles have also been employed as electrodes or electrocatalysts for fuel cells and batteries [16,17,18]. AgNPs have proven to be good catalysts for the oxygen reduction reaction (ORR) and its reversed version, the oxygen evolution reaction (OER) [19]. The ORR proceeds through different mechanisms, depending on the acidity/basicity of the medium and on the nature of electrodes promoting such reactions. Stabilizing or capping agents were found to have a significant effect on electrochemical properties of AgNPs.

At the core of the antimicrobial and electrocatalytic behavior of AgNPs lies the ability of these nanomaterials to perform oxygen-mediated redox reactions; in the case of antimicrobial materials this leads to an increase in the formation of ROS and the generation of silver ions [8,9,10]. In the case of electrocatalysts, such improvement facilitates oxygen reduction and oxidation reactions [19].

### 1.3. Potential for Enhancement of Electronic Properties of AgNPs

The electronic properties of AgNPs can be improved by modifying its composition, either at the core or at surface level. For example, carbon nanomaterials have been used with AgNPs without affecting their electrochemical properties [20,21,22]. Carbon nanomaterials have also been used to enhance the antimicrobial activity of titanium dioxide photocatalysts [23,24]. In particular, polyhydroxy fullerenes (PHFs) adsorbed on titanium dioxide have been shown to act as an electron relay and enhance photocatalysis [23,25]. Recently, PHFs have been employed as stabilizing agents for gold nanoparticles, and the nanoparticles generated have shown an increased catalytic activity towards phenol reduction [26].

Herein, we explore the possibility of using PHF as both a reducing agent and a stabilizing agent through a one-pot-synthesis method, and evaluate the ability of PHF to enhance both the antibacterial and electrocatalytic activity of silver nanoparticles.

## 2. Materials and Methods

### 2.1. Synthesis and Characterization of Citrate-AgNPs and PHF-AgNPs

Citrate-stabilized silver nanoparticles were synthetized through a variation of the Frens method [27], which has been previously reported by our group [7,28]. Briefly, 50 mL of silver nitrate 1 mM was placed in an aluminum foil-covered Erlenmeyer flask and heated to a boiling point. Then, 500 µL of a sodium citrate solution with a concentration of 0.189 M was added and the resulting solution was stirred for 20 min. The AgNPs that were synthesized were recollected by centrifugation at 2040 RCF for 30 min, resuspended in distilled water to a concentration of 33 mM, and stored at 4 °C in the dark. Polyhydroxy fullerene-coated silver nanoparticles (PHF-AgNPs) were synthetized by adding 5 µL of AgNO_3_ 1 M and 500 µL of PHF 2 mg/mL in an aluminum foil-covered vial and completing up to 1 mL with distilled water. Then, the solution was stirred at 20 °C for 4 h. Finally, PHF-AgNPs were recollected by centrifugation at 2040 RCF for 30 min, resuspended in distilled water to a concentration of 5 mM, and stored at 4 °C in the dark.

It is worth noting that even though different masses of silver were involved in these synthetic methods (~5 mg Ag for citrate-AgNPs and ~0.5 mg Ag for PHF-AgNPs), these differences were considered at the time the voltammetry and antibacterial experiments were performed, and comparable amounts of citrate-AgNPs and PHF-AgNPs were employed: concentration of AgNPs (in µg mL^−1^) in the antibacterial tests and mass of Ag NPs per square cm for electrodes in the voltammetric tests. The size and shape of the nanoparticles were characterized with high-resolution transmission electron microscopy (HR-TEM) using a FEI Tecnal (Hillsboro, OR, USA) F30 300 kV microscope). The hydrodynamic diameter was determined by dynamic light scattering (DLS) with a Wyatt Technology’s Möbiuζ^®^, Goleta, CA, USA; UV-visible spectroscopy (UV-vis) was performed using a NanoDrop^®^ ND-1000 spectrophotometer from Thermo Fisher (Waltham, MA, USA).

### 2.2. Antibacterial Assays

Antibacterial tests against *E. coli* (ATCC^®^ 8739) were performed following the procedures previously reported by our group [25]. Briefly, *E. coli* inoculated in 50 mL of Luria-Bertani (LB) medium for 24 h at 37 °C was purified and resuspended in the same medium. The resulting *E. coli* (8.8 × 10^9^ CFU mL^−1^) was diluted fivefold in LB medium. Then, 50 µL of the fifth dilution was suspended in 1 mL of distilled sterile water supplemented with citrate-AgNPs and PHF-AgNPs. As indicated above, in spite of the differences in the initial concentration of silver, the final concentration of nanoparticles in each sample was 500 µg mL^−1^. The resulting solutions were shaken for 5 min and 100 µL of the suspensions was cultured on MacConkey agar plates for 24 h. Finally, colonies were counted in each plate. All experiments were performed in triplicate.

### 2.3. Cyclic Voltammetry Tests

Cyclic voltammetry tests were performed in a three-electrode cell in alkaline medium using a ZIVE SP2 potentiostat (WonATech Co., Ltd., Incheon, Korea) with a scan rate of 0.02 V s^−1^. The reference and working electrode were, respectively, a mercury/mercury oxide electrode (E = 0.165 V vs. standard hydrogen electrode, SHE) and platinum. The working electrode was a conductive carbon nanotube (CNT) sheet (20 g.m^−2^ C-grade CNT Buckypaper, NanoTechLabs, Inc., Yadkinville, NC, USA), which was impregnated with nanoparticles through the ink evaporation impregnation method previously developed by our group. Briefly, citrate-AgNPs and PHF-AgNPs were resuspended in 800 µL of distilled water and 200 µL of isopropanol (2-Propanol, developed by J.T. Baker, TM, Madrid, Spain), and in 200 µL of distillated water and 50 µL of isopropanol, respectively. Then, the resuspension was sonicated for 30 min at 25 °C using a DR-S20 Ultrasonic Cleaner 3L (Shenzhen Derui Ultrasonic Equipment Co., Shenzhen, China). Next, 20 µL of the citrate-AgNPs resuspension and 50 µL of the PHF-AgNPs resuspension was put on the 1 cm^−2^ CNT electrode using 10 µL drops. The difference in volumes allowed comparable amounts of silver in both cases. The resuspension was evaporated using infrared light, leaving citrate-AgNPs and PHF-AgNPs impregnated in the CNT sheet. The resulting nanoparticle-based CNT electrode was wet with Nafion perfluorinated ion exchange resin before its immersion in the electrolyte, in order to overcome its hydrophobicity. The electrolyte was potassium hydroxide at 0.1 M. Several voltage windows, from −0.5 to 1.0 V, were employed to identify oxidation–reduction reactions corresponding to silver and oxygen. The average of nine cycles is shown.

## 3. Results and Discussions

### 3.1. Characterization of Citrate-AgNPs and PHF-AgNPs

**Figure 1:**
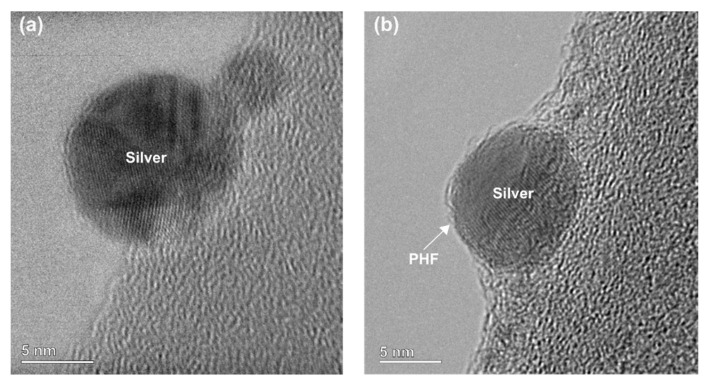
High-resolution transmission electron microscopy of citrate-AgNPs and PHF-AgNPs (**a** and **b**, respectively), showing in both cases ~10 nm particles. The PHF-AgNPs often had well-defined capping features surrounding them, which can be attributed to PHF molecules.

Silver nanoparticles in solution feature a characteristic yellow color [7], as seen in Figure S1 (Supplementary Material), for both AgNPs and PHF-AgNPs. For spherical nanoparticles, this color has been associated with nanoparticles of sizes below 20 nm [29]. Dynamic light-scattering (DLS) measurements made previously by our group [7,28] indicated that citrate-AgNPs have a hydrodynamic diameter below 10 nm, although greater sizes are also observed. High-resolution transmission electron microscopy (HR-TEM) images showed that both citrate-AgNPs and PHF-AgNPs are around 10 nm in diameter (Figure 1). It is interesting to note that HR-TEM images of PHF-AgNPs showed certain features surrounding the nanoparticles, which could be PHF molecules serving as capping agents (Figure 1b). UV-Vis absorption spectroscopy for citrate-AgNPs shows a broad peak around 418 nm, which is usual for spherical silver nanoparticles below 20 nm (Figure S2, Supplementary Material) [30]. Notably, the UV-Vis spectrum for PHF-AgNP exhibits a peak at 406 nm with narrower full width at half maximum (Figure S2), which indicates that the nanoparticle sizes are less dispersed. These results suggest that PHF is a reducing and stabilizing agent able to produce small AgNPs with a narrow size distribution.

### 3.2. Antibacterial Activity

**Figure 2:**
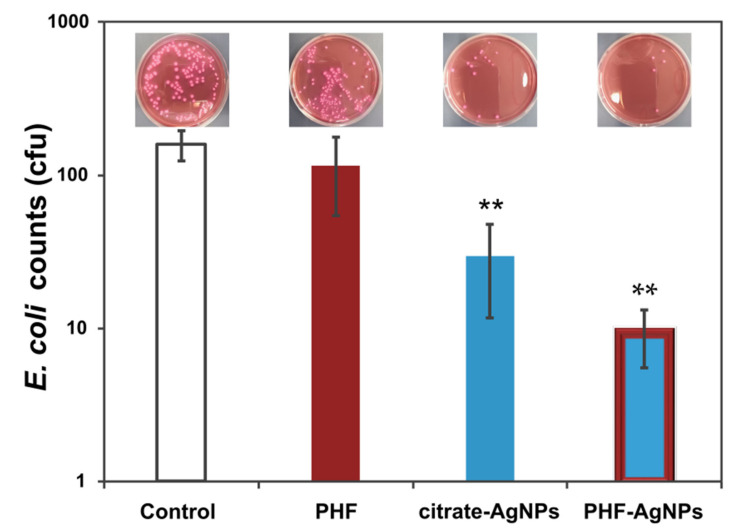
Examples of colony-forming units of *E. coli* in agar plates untreated (control) and treated with PHF, citrate-AgNPs, and PHF-AgNPs, after 24 h of culture. Notice that the cfu observed may differ from the values reported in Table 1 due to the fact that these values are the average of three antibacterial essays. PHF shows no antibacterial activity; however, PHF-AgNPs had higher antibacterial activity than citrate-AgNPs. As seen by the citrate-AgNPs and PHF-AgNPs bars (**), significant differences were observed with respect to the control experiment. This is not the case for PHF, which is statistically invariable with respect to control experiments.

Citrate-AgNPs and PHF-AgNPs were tested as antibacterial agents against *E. coli,* using comparable amounts of silver in both cases. The results of these studies are presented in Table 1 and plotted in Figure 2 to facilitate the comparison, together with selected views of test plates (notice that results in Table 1 are the average of three essays and the views correspond to the best results for each series). The antibacterial activity of citrate-AgNPs is evidenced by a decrease of 81.5% in the colony-forming units (cfu) of *E. coli* compared with the control sample. Experiments using PHF alone showed a very small reduction in bacterial counts, although not significantly different from the control. PHF-AgNPs exhibited the highest antibacterial activity, reducing 94% of the *E. coli* cfu. Further studies are needed to elucidate the specific mechanisms by which citrate-AgNPs and PHF-AgNPs are acting as antibacterial agents. It is possible that PHF, which is a better electron relay than citrate, could be enhancing the electron transfer process to aid in antibacterial activity [8]. Due to the relevance of electron transfer to electrocatalytic activity as discussed previously, we performed cyclic voltammetry in order to compare the catalytic activity of PHF-AgNPs with citrate-AgNPs.

### 3.3. Cyclic Voltammetry Test

**Figure 3:**
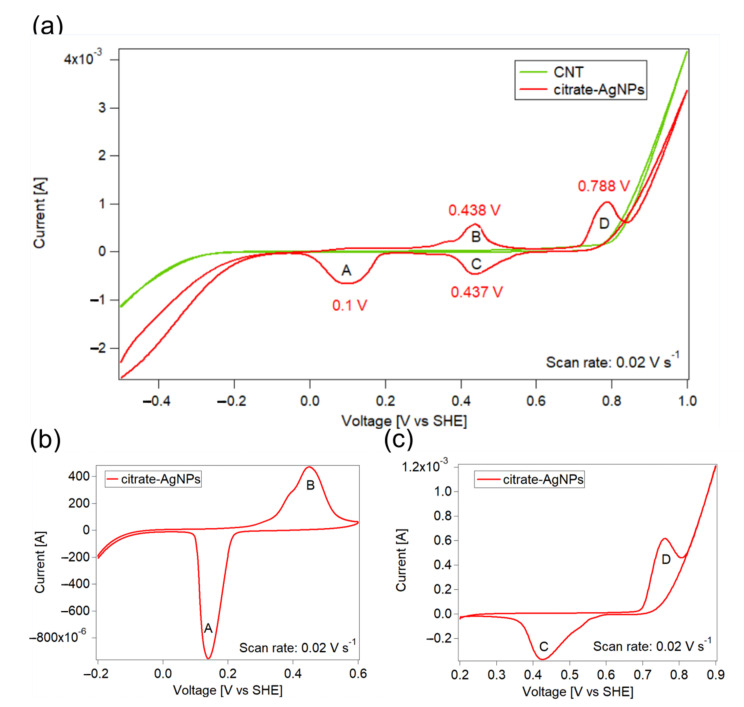
Cyclic voltammograms using citrate-AgNP-CNT electrodes. (**a**) Comparison with CNT electrodes (without silver), showing the 0.0–0.8 V window of the CNT electrode and the reactions promoted by citrate-AgNPs, peaks A through D; (**b**) voltammogram in the range −0.2–0.6 V, showing only peaks A and B, corresponding to silver reduction and oxidation peaks; (**c**) voltammogram in the range 0.2–0.9 V, showing only peaks C and D, corresponding to oxygen evolution and reduction peaks. The absence of peak C in (**b**) and of peak B in (**c**) indicates that the pairs C/D and A/B correspond to two independent redox reactions. In all cases, the scan rate was 0.02 V s^−1^ and the electrolyte used was 0.1 M KOH. Voltages are given with respect to the standard hydrogen electrode. There was no degassing using N_2_ nor another compound.1.

The catalytic activity of citrate-AgNPs and PHF-AgNPs was compared using the peaks shown in cyclic voltammetry. First, the silver and oxygen reaction peaks were identified using carbon nanotube-based electrodes decorated with citrate-stabilized silver nanoparticles (citrate-AgNP-CNT). Figure 3a shows cyclic voltammograms for CNT and citrate-AgNP-CNT electrodes. The CNT electrode does not produce any electrochemical reaction in the region from 0.0 to 0.8 V. Outside this electrochemical window, reactions corresponding to water reduction (below 0.0 V) and water oxidation (above 0.8 V) are observed. On the other hand, the citrate-AgNP-CNT electrode presents four peaks, named A through D in Figure 3a and centered around 0.1, 0.438, 0.437, and 0.788 V, respectively. In order to determine the corresponding reactions for each peak, cyclic voltammetry was performed in smaller windows. Figure 3b shows tests performed from −0.2 V to 0.6 V, where only peaks A and B were obtained, which means that these reactions correspond to a reversible reaction and shows that peak C can only be observed if peak D has been previously formed. This is confirmed by performing a voltammetric test using a 0.2–0.9 V window (Figure 3c). Thus, peak B is attributed to the oxidation of metallic silver to Ag_2_O and peak A is assigned to the opposite reaction (reduction of Ag_2_O into metallic silver) [31]. Similarly, peak C is attributed to the reduction of oxygen and peak D to the evolution of oxygen.

**Figure 4:**
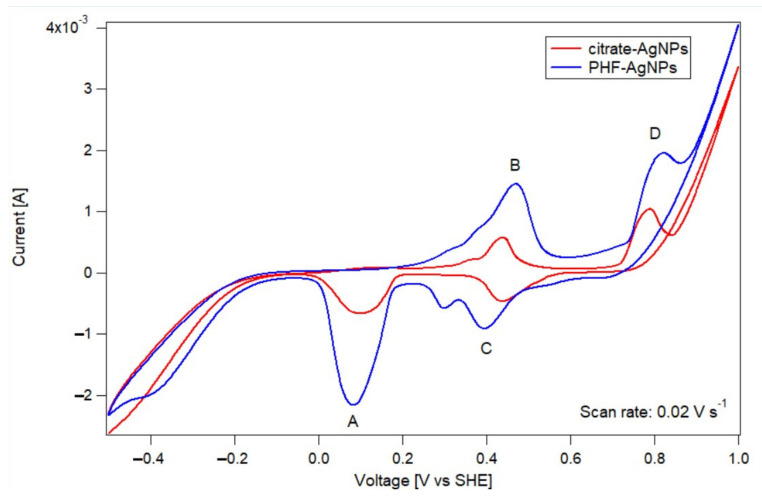
Cyclic voltammograms for the citrate-AgNP-CNT electrode (red) and the PHF-AgNP-CNT elec-trode (blue). In both cases, the scan rate was 0.02 V s−1 and the electrolyte used was 0.1 M KOH. Labeled peaks correspond to reduction of oxidized Ag (A), silver oxidation (B), oxygen reduction reaction (C) and oxygen evolution reaction (D), and show clearly an increased activity for the PHF-AgNPs with respect to the conventional citrate-AgNPs. Voltages are given with respect to the hydrogen standard electrode. There was no degassing using N2 nor another compound. Comparable amounts of silver are considered in both experiments.

Having identified the peaks corresponding to silver oxidation/reduction and to oxygen evolution/reduction, experiments using the PHF-AgNPs-CNT electrodes were conducted, as shown in Figure 4. It is possible to see a higher catalytic activity of PHF-AgNPs with respect to citrate-AgNPs, since the current intensity of PHF-AgNPs is much higher than that of AgNPs. This means that more oxygen and silver are reacting for the PHF-AgNPs-CNT electrode, even though comparable amounts of silver were employed in both cases. In addition, the onset potential of the ORR varies from 0.69 V for citrate-AgNPs to 0.81 V for PHF-AgNPs, which also demonstrates an increase in catalytic activity. Similarly, the onset potential of OER is 0.57 V for citrate-AgNPs and 0.53 V for PHF-AgNPs. Interestingly, in the case of the PHF-AgNPs, there is a smaller reduction peak at around 0.3 V. It is likely that this peak is due to an additional oxygen reduction reaction; however, the identification of this peak goes beyond the scope of this study and was not studied further. Experiments conducted using PHF-CNT electrodes (not shown) indicate that PHF by itself cannot facilitate OER and ORR. Thus, these experiments demonstrate that the unique electronic properties of PHF can be utilized to enhance the electrocatalytic activity of the silver nanoparticles.

## 4. Conclusions

Silver nanoparticles, around 10 nm in size with narrow size distribution, were successfully synthesized using, for the first time, PHF as a reducing and capping agent. Antibacterial essays with *E. coli* showed that PHF-AgNPs are highly effective, with similar or better activity than conventional citrate-AgNPs. Cyclic voltammetry tests showed that PHF increases the catalytic activity of silver nanoparticles towards OER and ORR. Since PHF by itself is incapable of promoting these electrochemical reactions, it is clear that this molecule acts synergistically with silver. Our results confirmed that PHF can be used as a single reducing and capping agent, and that PHF enhances the antibacterial and electrocatalytic properties of metallic nanoparticles, probably due to an enhanced electron transfer. Our study opens up various possibilities for catalytic and biomedical applications.

## Figures and Tables

**Table 1:** Summary data of essays of antibacterial activity of control, PHF, citrate-AgNPs, and PHF-AgNPs against *E. coli.* Data corresponds to *E. coli* counts, in colony-forming units (cfu).

Sample	Average	Standard Deviation	% Reduction
Control	160.0	35.6	--
PHF	115.7	61.2	27.71%
Citrate-AgNPs	29.7	18.0	81.46%
PHF-AgNPs	9.3	3.8	94.17%

## Data Availability

The data presented in this study are available on request from the corresponding author.

## Supplementary Materials

The following supporting information can be downloaded at: https://www.mdpi.com/article/10.3390/nano12193321/s1, Figure S1: Visual confirmation of silver nanoparticles in (**a**) citrate-AgNPs and (**b**) PHF-AgNPs. The silver nanoparticles have a typical yellow color, as has been previously reported. This color is present in (**a**) and (**b**), and is an indicator of the presence of silver nanoparticles in both colloids. In contrast, a PHF solution (**c**) has a brown color.; Figure S2: UV-visible spectroscopy of (**a**) citrate-AgNPs and (**b**) PHF-AgNPs. References [32,33] are cited in the Supplementary Materials.
