# Supplementary material for: Polyhydroxy Fullerenes Enhance Antibacterial and Electrocatalytic Activity of Silver Nanoparticles
Source: Nanomaterials (Basel). 2022 Sep 23;12(19):3321. doi: 10.3390/nano12193321 (PMC9565599; doi:10.3390/nano12193321)
Supplement: Supplementary file 1 [file nanomaterials-12-03321-s001.zip › nanomaterials-1893204-supplementary.pdf]

SUPPLEMENTARY MATERIAL FOR THE ARTICLE

Polyhydroxy Fullerenes Enhance Antibacterial and Electrocatalytic Activity of Silver Nanoparticles

Luis Palomino [1,2], Danae A. Chipoco Haro [1], Miguel Gakiya-Teruya [1], Feng Zhou [3], Adolfo La Rosa-Toro [4], Vijay Krishna [3] and Juan Carlos F. Rodriguez-Reyes [1,2,5,*]

1 Laboratory of Nanoscience and Applications—NASCA, Universidad de Ingenieria y Tecnologia—UTEC, 165 Medrano Silva, Barranco, Lima 15063, Peru
2 Centro de Investigacion en Bioingenieria—BIO, Universidad de Ingenieria y Tecnologia—UTEC, 165 Medrano Silva, Barranco, Lima 15063, Peru
3 Cleveland Clinic, Lerner Research Institute, Department of Biomedical Engineering, Cleveland, OH 44106, USA
4 Laboratorio de Investigacion de Electroquimica Aplicada, Facultad de Ciencias, Universidad Nacional de Ingenieria, Av. Tupac Amaru 210, Rimac, Lima 15333, Peru
5 Department of Chemical Engineering, Universidad de Ingeniería y Tecnología—UTEC, 165 Medrano Silva, Barranco, Lima 15063, Peru
* Correspondence: jcrodriguez@utec.edu.pe; Tel.: +51-94-912-7750

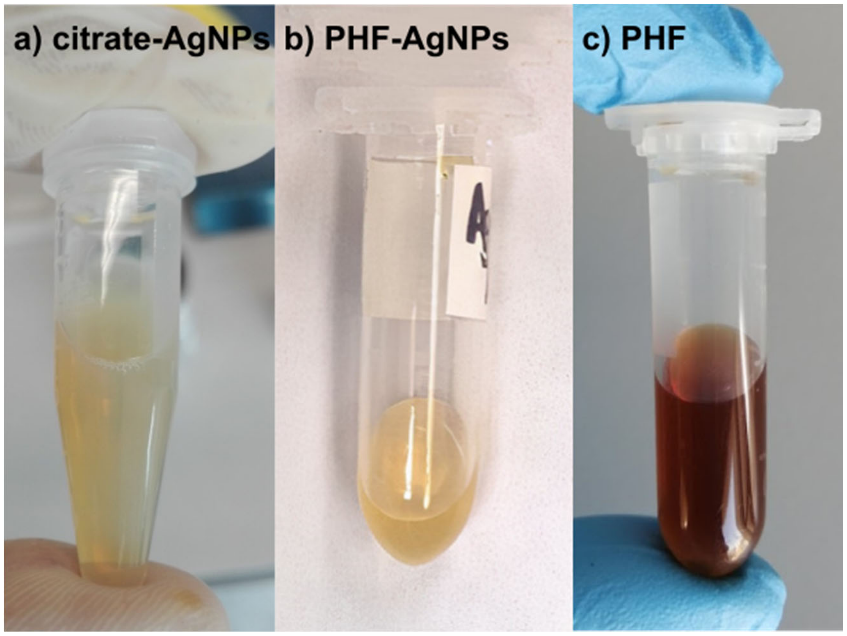

Figure S1. Visual confirmation of silver nanoparticles in (a) citrate-AgNPs and (b) PHF-AgNPs. The silver nanoparticles have a typical yellow color, as has been previously reported. This color is present in (a) and (b), and is an indicator of the presence of silver nanoparticles in both colloids. In contrast, a PHF solution (c) has a brown color.

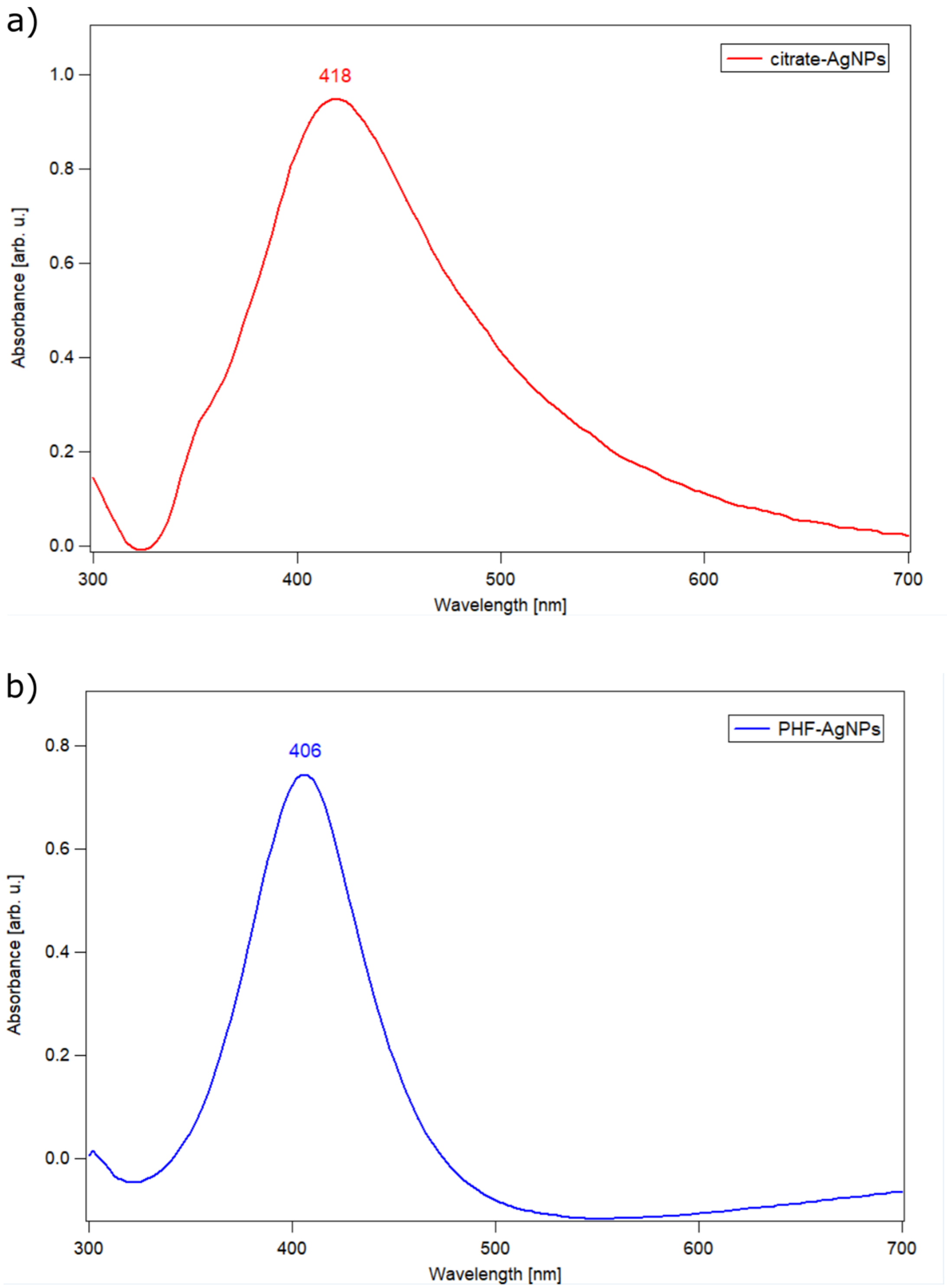

Figure S2. UV-visible spectroscopy of (a) citrate-AgNPs (red) and (b) PHF-AgNPs (blue). (a) Citrate-AgNPs show a broad peak at 418 nm, which is similar to previous reports for AgNPs (Judith Vijaya et al. 2017; Gakiya-Teruya et al. 2019; Rangayasami et al. 2021). (b) PHF-AgNPs show a peak at 406 nm with a narrower full width at half maximum suggesting more uniform nanoparticle sizes. Both spectra, (a) and (b), correspond to spherical silver nanoparticles below 20 nm.
